# International, Transdisciplinary, and Ecohealth Action for Sustainable Agriculture in Asia

**DOI:** 10.3389/fpubh.2021.592311

**Published:** 2021-02-05

**Authors:** Hung Nguyen-Viet, Giang Pham, Steven Lam, Phuc Pham-Duc, Tung Dinh-Xuan, Fang Jing, Pattamaporn Kittayapong, Wiku Adisasmito, Jakob Zinsstag, Delia Grace

**Affiliations:** ^1^Animal and Human Health Program, International Livestock Research Institute, Nairobi, Kenya; ^2^Center for Public Health and Ecosystem Research, Hanoi University of Public Health and Vietnam Public Health Association, Hanoi, Vietnam; ^3^Independent Research and Evaluation Consultant, Guelph, ON, Canada; ^4^National Institute of Animal Sciences, Hanoi, Vietnam; ^5^Institute for Health Sciences, Kunming Medical University, Kunming, China; ^6^Department of Biology, Faculty of Science, Center of Excellence for Vectors and Vector-Borne Diseases, Mahidol University at Salaya, Nakhon Pathom, Thailand; ^7^Faculty of Public Health, Universitas Indonesia, Jakarta, Indonesia; ^8^Department of Epidemiology and Public Health, Swiss Tropical and Public Health Institute, Basel, Switzerland; ^9^University of Basel, Basel, Switzerland; ^10^Natural Resources Institute, University of Greenwich, Kent, United Kingdom

**Keywords:** agricultural intensification, sustainable agriculture, food security, ecohealth, transdisciplinarity, Asia, health impact

## Abstract

Sustainably intensifying agriculture to secure food for people, while minimizing the human, animal, and environmental health impacts is an unprecedented global food security challenge. Action research is needed to understand and mitigate impacts, with Ecosystem approaches to health (Ecohealth) emerging as a promising framework to support such efforts. Yet, few have critically examined the application of Ecohealth principles in an agricultural context, particularly in Southeast Asia where agricultural intensification is rapidly expanding. In this paper, we evaluate the strengths, challenges, and opportunities of agriculture-related Ecohealth projects in low-resource settings of Vietnam, Thailand, Indonesia, and China, drawing on a case study of the Field Building Leadership Initiative (FBLI). To do this, we used a developmental evaluation framework involving several iterative cycles of document reviews, interviews, focus groups, and outcome harvesting with researchers, partners, and community members involved in FBLI. Results highlight the importance of transdisciplinarity, participation, and knowledge-to-action principles in co-generating knowledge and co-developing practical solutions. Implementing such principles presents challenges in terms of coordinating regional collaborations, managing high workloads, meaningfully engaging communities, and ensuring ongoing monitoring and evaluation. To address these challenges, there is a need to strengthen capacity in integrated approaches to health, improve institutionalization of Ecohealth, foster community engagement, and systematically monitor and evaluate efforts. Ecohealth holds significant promise in improving food security, but only when considerable time is spent developing and implementing projects with communities.

## Introduction

The 21st century presents us with some of the most profound global challenges including food and nutrition insecurity, climate change, emerging diseases, and antimicrobial resistance. Agriculture contributes to many of these but is also part of the solution. For example, agricultural intensification, or producing more food on less land, is an important strategy for meeting the rise in food demand by 2050, estimated to range from 60% upwards (1, 2). However, the concomitant use of water and modern inputs such as pesticides and fertilizers can lead to local environmental degradation (1, 3) along with public health risks to agricultural workers, community members, and consumers (4–6). More broadly, agricultural intensification processes have been linked to climate change exacerbation (7), zoonotic disease emergence (8), and antimicrobial resistance (9). The concept of “sustainable agriculture” implies raising yields while maintaining or bettering environmental, economic, and social conditions, but it is not yet evident how this can be achieved.

Agricultural systems in the Southeast Asia region are especially vulnerable to the trade-offs between food security and ecosystem functions. Comprised of 11 geographically, culturally, and politically diverse countries, the region as a whole is experiencing substantial agricultural growth, driven by population growth, international trade, and technological change (10). As nations rapidly develop, and agricultural processes quickly intensify, maintaining ecosystem resilience in the region will be challenging (11). Indeed, the region is already feeling the effects of resource use pressures and environmental change on food production and livelihoods, particularly in agroecological zones characterized by labor-intensive, rain-fed agriculture (12). Furthermore, the impending impacts of climate change and accelerating socioeconomic developments challenge food security in the Mekong Delta (13). Achieving the Sustainable Development Goal 2 of ending hunger, achieving food security and improved nutrition, and promoting sustainable agriculture by 2030 in Southeast Asia requires a better understanding of the intersection between human behavior, ecosystems, and agriculture (14).

The Ecosystem approaches to health (Ecohealth) framework is often considered a promising approach to address ecological sustainability and global health challenges (15, 16). Ecohealth recognizes that “health and well-being are the results of complex and dynamic interactions between determinants, and between people, social and economic conditions, and ecosystems” (16, p. 7). Six principles underline Ecohealth: systems thinking, transdisciplinarity, participation, gender and social equity, knowledge-to-action, and sustainability. In addition, the six principles by Charron (16) are not easily to fill in every Ecohealth project. That is why operational criteria were developed to assess Ecohealth projects (17) that requires at least two key principles of trans-disciplinarity and system thinking. In Southeast Asia, over 20 Ecohealth initiatives have been conducted (14). Because the region is a hotspot, projects primarily focused on emerging infectious diseases, which continue to be an important contributor to the burden of disease in low-resource settings. However, Ecohealth is not only concerned with disease ecology but also addresses wider environmental health concerns such as environmental contaminants, natural resource management, and agriculture.

While considered to have strong potential, few have critically examined the Ecohealth framework (18–20), particularly in the context of sustainable agriculture (21). Such critical reflections are essential if we are to learn from our experiences and to provide a foundation for future initiatives that aim to enhance ecological sustainability. In this paper, we evaluate the strengths, challenges, and opportunities of Ecohealth research and practice in rural agricultural community settings, drawing on the experience of the international transdisciplinary Field Building Leadership Initiative (FBLI). In doing so, we contribute regional perspectives from Southeast Asia which might be useful to other “regional chapters” engaged in Ecohealth (22).

## Methodology

### A Case Study of the FBLI Project

We focus on FBLI to develop insights into an agriculture-related Ecohealth program. FBLI was a 5-year (2011–2016) regional program comprised of Ecohealth projects in multiple countries. FBLI involved collaboration between the International Livestock Research Institute, Hanoi University of Public Health, Universitas Indonesia, Kunming Medical University, Mahidol University, World Agroforestry Centre, Vietnam Public Health Association, and Canada Vets without Borders. Core funding was provided by the Canada International Development Research Centre (4M CAD). FBLI aimed to (i) conduct Ecohealth research to understand and address the health risks of agricultural intensification; (ii) strengthen the capacity within Southeast Asia and China for Ecohealth research by institutional capacity building in Ecohealth; (iii) engage key policymakers to ensure that emerging research findings inform policy and practice in the field; and, (iv) facilitate knowledge sharing at national and regional levels to mainstream Ecohealth and foster the development of the Ecohealth field in the region.

**Figure 1:**
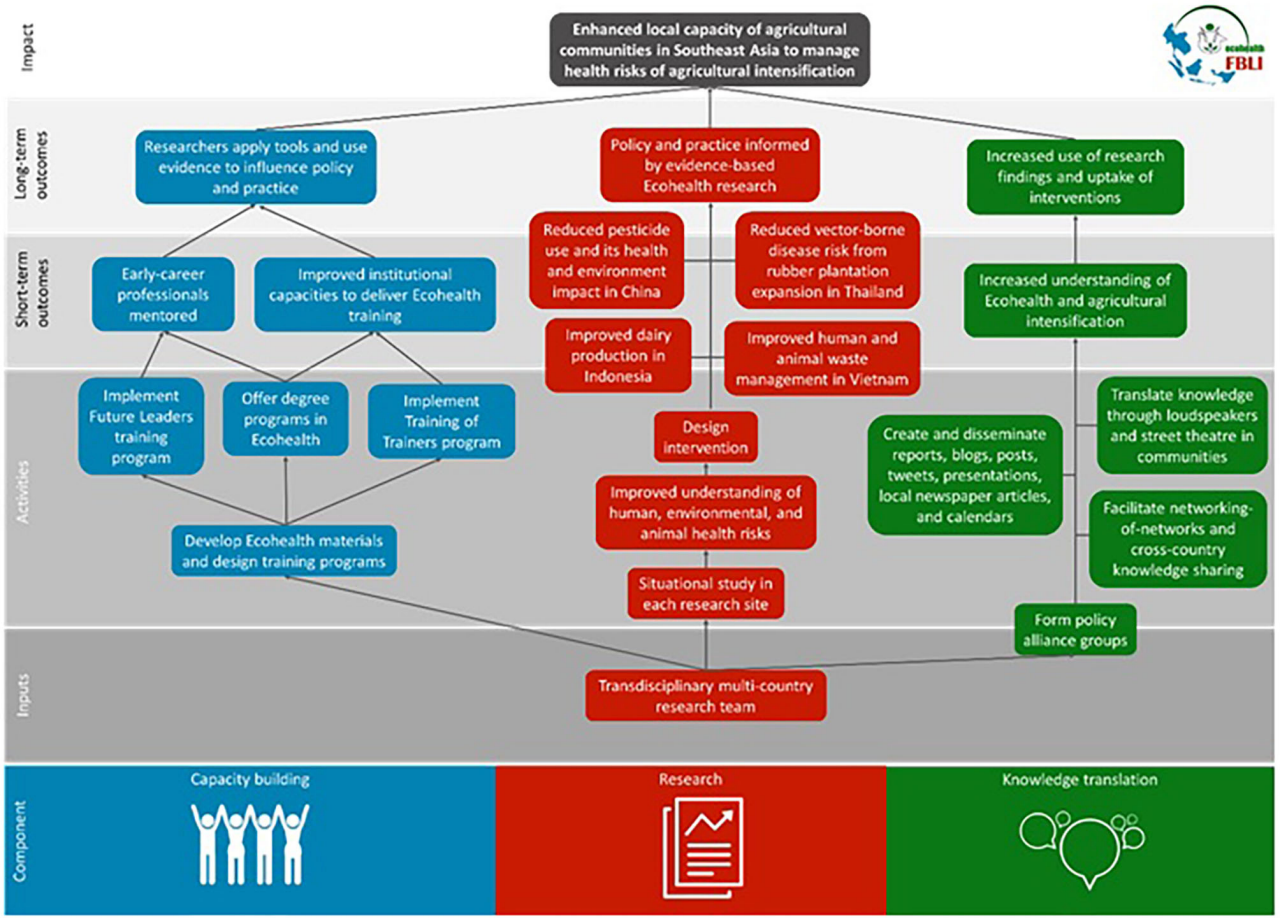
Impact pathway of Ecohealth projects in China, Indonesia, Thailand, and Vietnam.

FBLI objectives were achieved by carrying out three interlinked components: research, capacity building, and knowledge translation. FBLI used a site-based approach to research. The site-based approach involved focusing on a specific location with a strategic purpose, rather than focusing on a particular health problem, allowing for an open perspective and long-term engagement with local communities. Research activities generated evidence to develop knowledge translation materials and inform policy. Also, the research component provided study sites for young scholars and students to train in Ecohealth. It also provided teaching materials to support the development of training curriculum and programs targeted at different audiences in the region. See Figure 1 for the program impact pathway depicting how FBLI aimed to attain its goals.

Using an Ecohealth approach, FBLI brought together an interdisciplinary team of university-based faculty, NGO-based researchers, and students. The team comprised epidemiologists, medical doctors, public health specialists, veterinarians, anthropologists, and agricultural economists. Faculty researchers based in the country led the project in the country (Author initials: W.A., P.P-D, F.J., P.K.). A coordinating unit based in Vietnam was responsible for project management and evaluation (H.N-V., G.P., T.D-X). Once an initial Ecohealth training workshop was conducted, various ways helped ensure ongoing exchange, learning, and collaboration. For example, additional workshops were held. In our regional knowledge translation workshop during our 5th year, teams developed policy-relevant key messages from Ecohealth work conducted in the four FBLI countries. In our annual face-to-face meetings, each country presented their findings and lessons learned. Additional meetings were held to provide country updates on progress and challenges.

The team working closely with rural agricultural communities with sites in Vietnam (Ha Nam Province), Indonesia (West Java), China (Yunnan Province), and Thailand (Chachoengsao Province), and with decision-makers at local to national scales. Sites were selected based on several criteria, including agricultural intensification, the cooperation of local stakeholders, accessibility to the research team, and potential for scaling-up. All four sites first conducted situational analyses to characterize the complex interactions between agricultural practices, the biophysical environment, and the social, economic, and cultural context. The situational analysis identified the following research priorities: safe pesticide use in China; human and animal waste management in Vietnam; rubber plantations and vector-borne diseases in Thailand; and small-scale dairy farm waste management in Indonesia. To examine the health risks of agricultural intensification, teams conducted a variety of research activities depending on the research problem, including participatory rural appraisals, cross-sectional surveys, interviews, focus groups, laboratory testing, and disseminating and promoting the use of findings.

### Evaluation Approach

Agricultural sustainability in Southeast Asia and China is a complex challenge, often characterized by a plurality of perspectives, a multifaceted nature, and a diverse socio-political context. Furthermore, agriculture can be seen as a complex system comprising physical, cultural, and economic dimensions, which influence (and is influenced by) development programs. Considering the complexity of the agricultural intensification problem in Southeast Asia and China, the uncertainty in the types of outcomes achievable by an agriculture-related Ecohealth program, and the need to support program learning and adaptation, a developmental evaluation approach was chosen. Developmental evaluation is well-suited to assess programs implemented under dynamic environments in which multiple influences make it difficult to predict what will happen as a program proceeds (23, 24). Developmental evaluations track outcomes as they emerge and provide real-time feedback for program improvement. Typically, in developmental evaluations, the evaluator is positioned with a project team. A benefit of having the evaluator (in this case, the coordinating unit) embedded in the project team, is that timely feedback could inform decision-making at the broader program level. This was influential in selecting a developmental approach.

A key tenet of developmental evaluation is iterating cycles of reflection. In our developmental evaluation approach, evaluation was conducted with the same participants during the 5-year program cycle to gather and respond to feedback. Depending on the progress that teams made in their country-specific project, either two to three cycles of evaluation were conducted with the team. The coordinating unit led the data collection process in each region, emphasizing confidentiality in this process to ensure an open space for reflection and dialogue. By engaging with the same participants at each cycle, the coordinating unit could systematically document the process of change in participants' actions, behaviors, and attitudes toward the Ecohealth program.

### Evaluation Methods

Each iteration of the developmental evaluation consisted of document reviews, interviews, focus group discussions, and outcome harvesting. Documents reviewed included technical reports, meeting notes, peer-reviewed publications, and field notes. These documents provided information about the context and scope of individual country projects, along with insights into stages of project development and implementation. The documents were used to identify issues, track key decisions, process, and outcomes, and plan for field visits to conduct interviews, focus group discussions, and outcome harvesting sessions. Across all data collection methods, our discussions were open-ended rather than structured around predetermined criteria to understand what participants valued. In this way, quality is assessed by nature of outcomes expressed by participants and the extent to which FBLI contributed to outcomes.

A total of 38 interviews with community members and other decision-maker partners were interviewed over the 5-year program in this study. The interviews were semi-structured and conversational. Specific questions varied by country and type of participant. All interviews were confidential and carried out in the participants' preferred language with research assistants providing translation where needed. We asked participants to reflect on: changes in their behaviors, attitudes, actions, and relationships from involvement in the Ecohealth program; what worked and what did not; and any unexpected outcomes.

We adapted and conducted outcome harvesting (*n* = 11) with research team members including students, staff, NGO-based researchers, and faculty-based researchers of the FBLI program to identify outcomes both within and outside the team. Outcome harvesting focuses on the contributions of programs to outcomes, broadly defined as changes in the behavior, relationships, activities, or actions of the people with whom a program works directly (25). The method first examines evidence on what has been achieved; then, works backward to determine whether and how the program contributed to the change. To ensure feasibility in repeating this activity several times, we conducted rapid outcome harvesting sessions consisting of: [1] reviewing program documents and formulate outcome descriptions; [2] discuss with participants about the outcomes; [3] revising outcome descriptions; [4] substantiating information against the documents, interviews, and focus group discussions; and, [5] disseminating and supporting the use of findings.

### Analysis

Thematic analysis using a constant comparative method was used to analyze the data (26). All transcripts were transcribed by two researchers having sole access to transcripts and identifiable data (G.P. and S.L.). Transcripts were initially read in full; next, segments of the text were coded and organized into categories. Within the categories, the codes were analyzed and summarized into key themes on the strengths, challenges, and opportunities of Ecohealth programs extracted. To validate results, emerging findings were discussed with the research team, community members, and other decision-making partners, and also compared to findings from other studies evaluating Ecohealth programs.

## Results

The design and implementation of Ecohealth projects varied given the different country priorities around agricultural intensification (see Table 1 for an overview of the Ecohealth projects).

**Table 1:** Overview of Ecohealth study sites, objectives, research, and interventions in China, Indonesia, Thailand, and Vietnam.

**Component**	**Countries**			
	**China**	**Indonesia**	**Thailand**	**Vietnam**
Study site	Yuanmou County, Yunnan Province	Pangalengen, West Java	Chachoengsao Province	Hanam Province
Context	- Vegetable plantation is an economic development priority of the local government - Pesticides and fertilizers are often used in Yuanmou County	- West Java is the second largest milk producer in Indonesia - Significant challenges remain for the development of smallholder dairy farms	- Thailand is the largest producer of rubber, largely due to government promotion of rubber expansion - Land transformations are impacting malaria transmission	- Livestock production is rapidly increasing due to growing demands for meat - The management of farm waste is not keeping up with agricultural development
Objectives	- Examine the history, current situation, drivers, and future trend of pesticide use - Determine the impact of pesticide use on human and environmental health - Develop interventions to promote sustainable pesticide use	- Identify issues of underperforming smallholder dairy farms in Indonesia - Develop interventions that will have a positive impact on animal, environmental, and human health and economic profitability of smallholder dairy farms	- Examine the ecology of vectors and vector-borne diseases in rubber plantations - Determine the relationship between ecological, biological, and social factors of rubber plantations and their implications on vector-borne diseases	- Examine the health risks of human and animal waste management - Characterize socio-economic and cultural factors surrounding waste management - Develop interventions to improve waste management
Research	- Field site visits - Secondary data analysis - Interviews with health workers, pesticide sellers, farmers, and local government representatives - Survey of 418 farmers and 298 plantation farmers - Pesticide residue testing in vegetables, water, and urine	- Field site visits - Secondary data analysis - Interviews with farmers and community leaders - Focus group discussions with farmers - Survey of 148 farmers - Testing of agricultural products - River water sampling	- Field site visits - Secondary data analysis - Interviews with rubber plantation owners, managers, and workers - Focus group discussions with rubber workers - Survey of 84 rubber workers - River water sampling	- Field site visits - Secondary data analysis - Focus group discussions with community members and local authorities - Interviews with farmers, health workers, and local authorities - Survey of 451 households - Wastewater sampling
Intervention	- Training workshops with farmers on the correct use of pesticides and personal protective equipment - Sharing urine test results to farmers face-to-face to provide education - Educational campaigns in the community (street theater, calendars, posters)	- Development of four reusable products from animal waste (biofertilizer, earthworm fees, organic fertilizer, animal herbal feed supplement) - Formation of a business incubator to facilitate product commercialization - Providing on-farm management training	- Development of insect repellent jackets for workers - Suppression of vectors using super-sterile *Aedes aegypti* male mosquitoes - Media engagement and community meetings to provide knowledge about mosquito birth control	- Training a core group of farmers and promoting peer-to-peer sharing in the community - Workshops with a biogas expert - Modified and promoted the traditional village document around environmental sanitation - Education campaigns in the community (posters, calendars, loudspeakers, booklets)

### China

Since 1980's, vegetable plantations have been greatly promoted by the local government in Yuanmou County, which has been accompanied by increases in the use of pesticides and chemical fertilizers. Although the county had switched to “low toxic” pesticides in the 2000's, FBLI-China research found that local people were still exposed, with more than 60% of urine samples collected testing positive for at least one kind of pesticide. The local environment was also found to be polluted: 40% of soil samples and 22% of water samples tested positive for at least one type of pesticide. Farmers highly depended on pesticides in the commercial vegetable plantation but most of them did not know how to choose and use pesticides properly. More farmers started using self-protection measures resulting in a reduced positive rate of pesticide residues in urine, due to the influence of health education campaigns by the team. Of note, farmers did not reduce the use of pesticides. The excessive use of chemical pesticides in agricultural production is caused by a complex set of interactions between different actors with diverse interests that are deeply embedded in agricultural development policy. Policy aiming at agricultural modernization and intensification led to heavy dependence on a new variety of seeds, planting techniques, and agricultural inputs. Without tackling these complex underlying drivers, “it is difficult and also unfair to ask farmers to reduce pesticide using.”

### Indonesia

Smallholder dairy farming is an important source of livelihood for rural people in Indonesia, yet significant challenges remain in its development, including low productivity, a high price of quality feed, and poor sanitation. FBLI-Indonesia research aimed to characterize farming activities of smallholder dairy farms in Pangalengan, in the highlands of West Java, and identified poor farm management as a key factor influencing the low quality of dairy products. Furthermore, river water samples were collected, analyzed using several water pollution indicators, and compared to Indonesian water quality standards. The team found that livestock activities affected water quality, posing health risks to livestock, humans, and the environment. To reduce the impact of river contamination, the team developed ways to convert cow waste into fertilizer and other products for recycling and reuse purposes. Preliminary on-farm trials suggested these products could provide a new source of income for farmers, increase crop yields, and reduce impacts on the environment.

### Thailand

Rubber plantation is an important commercial crop in Thailand, creating employment and income generative activities for local people. Yet, rubber plantation is also known to be a significant site for malaria transmission. The FBLI-Thailand team used the changing risks of vector-borne diseases as an entry point to explore how the rapid expansion of rubber plantations had an impact on the health and livelihoods of communities in eastern Thailand. Results revealed that risks for dengue, chikungunya, and malaria appeared to be higher in areas with rubber plantations in comparison to those without rubber plantations. Rubber workers spent most of their time, day and night, working in the rubber plantations which made them more vulnerable to vector-borne diseases due to prolonged exposure to mosquito vectors that were abundantly present. Also, environmental contaminations caused by inappropriate use of chemical fertilizers were observed. Rubber workers and owners of rubber plantations became more aware of their health and the influence of the environment, in part due to health education on self-protection of vector-borne diseases as well as best practices for chemical use promoted through many interactions with the team. Furthermore, community members improved the knowledge of mosquito vector control through community and media engagement. DEET-impregnated screen jackets and super-sterile male mosquitoes were developed during the project as short-term and long-term interventions, respectively.

### Vietnam

In Vietnam, livestock waste is commonly reused in agriculture as fertilizer, contributing to environmental sustainability and economic activity. However, increased livestock waste combined with often outdated management practices can present human and environmental health risks. The FBLI-Vietnam team explored waste management practices in Ha Nam Province and found the use of biogas systems to treat livestock waste was common practice in smallholder farms. Such systems provided gas for cooking while the effluent was used for crop irrigation. However, water sampling results revealed the concentration of pathogens in biogas effluent was high. The biogas effluent exceeded standards for *E. coli, Salmonella, Giardia*, and other harmful contaminants. Biogas effluent used as fertilizer also placed farmers at high risk of diarrhea. A core group of 12 farmers worked with the research team to develop and implement campaigns focused on proper biogas management. These campaigns, together with peer-to-peer communication, contributed to improved knowledge and practice of farmers in using household biogas. The quality of biogas effluent also improved as a result of safer biogas use.

### Cross-Country Findings and Development of Locally-Relevant Interventions

The situational analysis involved engaging communities, local authorities, and multi-sector stakeholder groups in discussions around potential interventions. This process led to the development of locally-relevant intervention packages. Interventions varied widely, from vector control technologies in Thailand to the development of reusable waste products in Indonesia. Both Vietnam and China focused on socially and culturally appropriate information, education, and communication materials. While this diversity in topics limited our ability to conduct a comparative analysis, we find several common elements in the four countries: [1] the development of interventions with communities; [2] the use of transdisciplinarity, participation, and knowledge-to-action principles of Ecohealth; and [3] the assessment of the intervention packages on health (all countries), environment (all countries), and animals (Thailand). The different Ecohealth projects also provided opportunities for cross-country learning as agricultural issues were not unique to one country. For example, research from FBLI-China helped inform student projects in Indonesia and Vietnam where pesticide use was identified as a concern.

### Advancing Scholarship on the Intersection of Agriculture and Health

**Figure 2:**
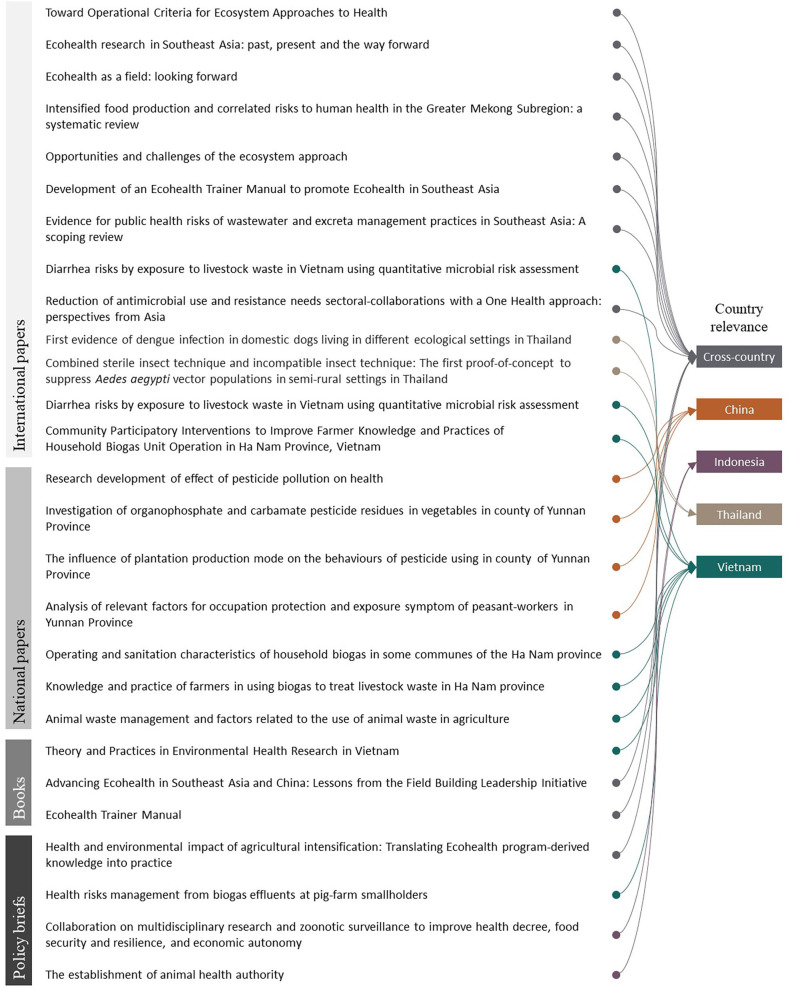
Publications from Ecohealth projects in China, Indonesia, Thailand, and Vietnam.

Advancing scholarship was considered an important contribution of agriculture-related Ecohealth projects by the research team and decision-making partners across countries. FBLI was viewed to have made substantial contributions to knowledge by demonstrating that pesticide use, rubber plantation expansion, and waste management present substantial risks to human health. Despite inherent challenges in conducting high-quality research in resource-limited settings (27), science outputs were produced by all four countries working in dynamic environments and addressing different research problems. A total of 13 international papers, seven national papers, four local policy briefs, and three books were published (Figure 2). At the regional level, a synthesis booklet and policy brief were also developed. These outputs contributed to sustainable intensification by explicitly addressing the negative externalities of intensification (such as increased pesticide use) and suggesting how they could be mitigated. By contributing to scholarship, FBLI improved the global visibility Ecohealth as well as health-related agricultural challenges. The efforts of FBLI were recognized internationally, with two members of FBLI receiving an award (Outstanding Contributions to Ecohealth; Fang Jing, 2014) and (Exceptional Early Career Award; Hung Nguyen, 2016) from the International Association for Ecology and Health.

### Improving Research Capacity Through Transdisciplinarity and Participation

For many researchers, community members, and decision-maker partners, transdisciplinarity represented a new way of conducting research. A transdisciplinary approach “integrates different scientific perspectives and provides a formal platform for stakeholder participation in the research and the development of new information, ideas, and strategies, their testing, and eventual application” (16, p. 11). To illustrate, the FBLI China team worked frequently with local villagers and community partners to determine and address agricultural challenges, identifying pesticide risks and safe use of pesticides as research priorities. A team member reflected: “While in traditional research, people just come once or twice during data collection. [The community partner] sometimes felt annoyed when [FBLI researchers] came back very often.” The team member added that this iterative participatory process had fostered relationship-building and a deeper understanding of Ecohealth research among groups. Participation in the regional partnership was also noted as instrumental in supporting learning between research teams. Through annual face-to-face regional core meetings held in each country, team members had an opportunity to share project-level learnings and work together to advance regional-level goals.

Beyond the boundaries of FBLI research, FBLI supported capacity building through the development of an Ecohealth trainer manual. This manual informed an Ecohealth textbook published in Vietnamese, and thus, helped to build Ecohealth capacity in Vietnam. FBLI efforts also led to the institutionalization of integrated approaches to health. At Mahidol University, an MSc and PhD training program titled “One Health and Ecosystem Management” was developed. Moreover, Ecohealth curricula have been integrated into all four FBLI partner universities. FBLI offered 20 small seed research grants to the four countries, providing opportunities for young researchers to apply the Ecohealth framework in solving community-identified challenges. Finally, FBLI developed a Future Leaders program, a series of training for future leaders in global health. This program was conducted in all four FBLI countries with over 200 students and professionals participating from 10 countries. These efforts contributed to sustainable intensification by introducing the concept to a wide range of researchers, decision-makers, and community members.

### Knowledge-to-Action Toward Sustainable Agricultural Practices

The research evidence generated by FBLI helped inform knowledge translation efforts and community-level interventions, which ultimately shaped the health practices of farming communities. Through knowledge translation efforts (local newspapers, loudspeakers, street theater, calendars, and posters), FBLI improved knowledge of, and interest in the impact of agricultural intensification on health among community members and decision-makers. To illustrate using the Vietnam case study, the promotion of research findings through posters prompted local authorities to target environmental sanitation (e.g., excreta, wastewater, and solid waste management; drainage and water supply) in rural development plans [see (21) e.g., of posters]. In Thailand, FBLI research findings were shared in 64 news articles, 20 TV programs, and three radio programs. By engaging local villagers and authorities in the co-design and co-implementation of interventions, FBLI also contributed to changes in sustainable agricultural practices among community members across countries, with the extent varying across countries. Farmers in China adopted personal protection measures when spraying pesticides on vegetables, reducing the positive rate of pesticide residues in urine; rubber workers and owners of rubber plantations in Thailand were more aware of how the environment may affect their health, in a context where vector-borne diseases were not a concern; pig farmers in Vietnam adopted safe use of biogas systems for waste management, resulting in cleaner biogas effluent for the environment; and, farmers in Indonesia developed bio-fertilizers from cow waste, which not only contributed to reducing environmental burden but also provided a new source of income for farmers.

## Discussion

Increasing interest in Ecohealth projects is part of a broader trend to consider the health of the environment, animals, and humans together. Despite its strong rationale and the increasing popularity of Ecohealth programs, there has been little reflection on the strengths, challenges, and opportunities of applying Ecohealth principles to a sustainable agriculture context. We contribute to a nascent body of literature critically examining the Ecohealth framework by sharing the experiences of researchers, community members, and other decision-maker partners engaged in an international transdisciplinary agriculture-related Ecohealth program. While overall the views of the Field Building Leadership Initiative (FBLI) were positive, themes concerning regional collaboration, workload demands, community engagement, and monitoring and evaluation were emphasized across countries, thus offering broad insights to assist researchers working in similar contexts.

### Regional Collaboration

From a project management perspective, facilitating and maintaining collaborations between country teams was particularly challenging. Similar to the experiences of Nordhagen et al. (28), we found that implementation often took on the form of “collaboration” rather than “integration,” whereby countries focused on their topics with limited cross-country responsibility. Communication between country teams substantially reduced from year three onwards, and country teams was did not have the same level of responsiveness as they did during the proposal development phase or the beginning of the project implementation phase. This decrease in regional collaboration toward the latter stages of FBLI could be explained by the competing time commitment required by intervention implementation. A strategic plan for regional collaboration would have helped FBLI manage these challenges by clearly outlining collective goals and timelines for achieving goals. Furthermore, more team meetings, either in-person or via teleconference, would have supported engagement. Indeed, team-building efforts were noted to be especially important for regional collaboration and collective learning, including annual regional meetings rotating between the countries and research, workshops, and cross-country mentorship. As noted by Cole et al. (29), implementers of multisector programs must plan for things to take longer and plan for cross-sector integration. Like other scholars (14, 19, 30), we call for more regional Ecohealth approaches and plans for regional collaboration to reach collective goals.

Institutions and administrative processes also created barriers for cross-institutional collaboration. In our view, administration tends to be inflexible, whereas Ecohealth research is flexible. Changes in research plans, as often occurring in Ecohealth research, complicate administrative processes which lead to delays. In some instances, research team members were prohibited from attending meetings and workshops abroad. Institutions should recognize that Ecohealth is a new terrain of research and practice, that is flexible and holds significant potential in improving the ways we do research. The institutionalization of Ecohealth teaching is important for advancing the field, recognizing that the challenges of doing so will lessen as we approach a paradigm shift where transdisciplinarity, participation, and knowledge-to-action become the new research norm. We also call on governments and global/regional bodies in Southeast Asia – such as Global Health Security Agenda (GHSA), Association of Southeast Asian Nations (ASEAN), Food and Agriculture Organization of the United Nations FAO, and World Health Organization (WHO), United States Agency for International Development (USAID) – to mainstream Ecohealth approaches into regional and national agricultural policies.

### High Workload Demanded by Ecohealth Principles

Related to the above challenge is the considerable time and resources demanded by Ecohealth principles, particularly the need for systems thinking and covering a broad range of research issues. It was challenging for researchers to balance knowledge production, intervention development, and advocacy, requiring researchers to make trade-offs between breadth and depth. Researchers reported having to wear multiple hats including a facilitator, coordinator, leader, and advocate, which is often uncomfortable for those that are not experienced in such roles. Our experiences lead us to the question: what are the boundaries of an Ecohealth project? We invite researchers working at the intersection of human, animal, and environmental health to grapple with this question.

The experiences of FBLI highlighted several opportunities to manage these tensions, one of the most important being the need to develop Ecohealth capacity among the research team. FBLI focused more-so on Ecohealth capacity building institutionally rather than individually. Consequently, researchers felt they did not have enough time to fully understand the Ecohealth principles, which were considered new ways of researching by most of the team members involved. By focusing first on building Ecohealth capacity among the research team, research teams would have had foundational knowledge and skills to better handle the demands of Ecohealth principles. Another strategy would be to prioritize certain Ecohealth principles, which appeared to be the case here as only three principles were largely emphasized by participants.

Considering the existing demands of Ecohealth principles on research and interventions, FBLI was not able to fully explore select principles including systems thinking, and equity sustainability. In these case studies, research and interventions mainly focused on human health. The consideration of ecosystem and animal health was not well-addressed. It was noted that such considerations were largely outside the scope of projects and the capacity of researchers who come from health backgrounds. Furthermore, recognizing that economic opportunities in agriculture are important for improving health outcomes and livelihoods, FBLI aimed to conduct economic analyses of agricultural interventions. However, due to constraints in time and resources, economic analyses were not done. As with economic considerations, gender and social equity mainstreaming were also planned in the initial design stages, but the depth of gender and social analysis conducted ended up being minimal. Finally, although longer-term uptake was not probed in our evaluation, we postulate that outcomes will be maintained given the gains made in institutionalizing Ecohealth training as a result of FBLI efforts. For future projects, we recommend partnering with more experts in the fields of gender, agriculture, livestock, and the environment.

### Community Engagement

Researchers initially faced challenges in community engagement, likely because it was a new approach for most. Researchers spent a considerable amount of time working and living in communities to build trust, relationships, and an understanding of community needs and assets. As the relationship between the research teams and communities became more established, communities became motivated to participate in research and interventions. It was highlighted that building the capacity of community members as co-researchers also took extensive time, with the extent of community engagement and progress varying between country teams. Yet, such efforts were important to improve the knowledge, attitudes, and practices of farmers and other concerned stakeholders, and thus, improve the management of health risks from agricultural intensification.

Researchers looking to apply Ecohealth principles should not underestimate the time required to build relationships and raise awareness of certain local issues. For instance, in Vietnam, local people were concerned with pollution but smallholder livestock farmers and local authorities were not so interested. Time and energy were required to gain the support of these two stakeholder groups. Time and resources were needed to train community members in engaging in the Ecohealth process, which was also reported by Chimbari (20). Furthermore, given the history of extractive research in many communities we worked in where researchers collected data without sharing them (31), it is important to provide community members with research findings. In China, for example, urine test results for pesticide residues were returned to community members face-to-face, providing an opportunity for relationship building and knowledge sharing.

### The Role of Monitoring and Evaluation

The developmental evaluation approach reported here was viewed as an important activity among research teams. It had a central role in capturing outcomes, fostering learning and reflexivity, increasing communication between country teams, and informing decision-making at the broader program level. For example, the outcome harvesting sessions encouraged teams to reflect on their contribution and learn from their experiences. By then sharing their reflections at the regional program level, other research teams could learn what was possible in Ecohealth research and practice, thus providing a source of motivation for individual country projects. However, monitoring and evaluation could have been conducted more frequently to address the emerging challenges raised here, considering programs are dynamic and not static entities (32). To achieve this requires a significant budget line and dedicated staff. Our evaluation was led by a program coordinator with many other roles, as well as an external consultant engaged toward the end of the program. We recommend that country teams conduct, document, and share self-reflective exercises at key milestones to support monitoring and evaluation efforts.

## Conclusion

This paper examines the strengths, challenges, and opportunities for conducting Ecohealth projects with rural agricultural communities in Southeast Asia and China, drawing on the experiences of the international transdisciplinary Field Building Leadership Initiative. This work suggests Ecohealth principles, particularly transdisciplinarity, participation, and knowledge-to-action, can help communities understand and address the impacts of agricultural intensification on health. Many in the communities adopted safe agricultural practices related to pesticide use, rubber cultivation, and waste management. Decision-maker partners gained knowledge of various health challenges facing communities as well as skills to participate in collaborative projects. Also, there was some evidence of reduction of the harmful impacts of agricultural intensification on environmental and animal health.

The paper also uncovered the complex nature of applying Ecohealth principles in the context of agriculture in low-resource settings of Vietnam, Thailand, Indonesia, and China. For instance, we found challenges in coordinating regional collaboration, balancing various roles, engaging with communities, and ensuring ongoing monitoring and evaluation. In our view, Ecohealth holds substantial promise in advancing food security, but only when considerable time, energy, and resources are spent focusing on project development. Through many iterations of project development with agricultural communities, local authorities, and other decision-maker partners, the pathways toward sustainable agriculture can be made clearer. While FBLI has concluded since 2016, team members have been continuing to apply Ecohealth principles to relevant projects, without necessarily labeling their work “Ecohealth,” similar to other Ecohealth practitioners (33). Also thanks to Ecohealth and One Health movement in Southeast Asia and China for nearly two decades, the transdisciplinary research including the use of socio-economic approach has been taking place more often. Young key members from FBLI have grown in their career and are holding important positions in their institution to promote Ecohealth activities nationally and regionally. The group of FBLI has been continuing to maintain their network and explore common projects in the region. Although FBLI has made substantial progress in mainstreaming Ecohealth into institutions, research, and practice, continued investments in Ecohealth is important for ensuring the sustainability of the concept in Southeast Asia and China.

## Data Availability Statement

The raw data supporting the conclusions of this article will be made available by the authors, without undue reservation.

## Ethics Statement

The studies involving human participants were reviewed and approved by Ethical approval for this research was obtained from Hanoi University of Public Health on 19 February 2013 (Decision No 041/2013-HD3). The patients/participants provided their written informed consent to participate in this study.

## Author Contributions

HN-V and SL designed and wrote the manuscript. All authors read, commented, and agreed on the submitted manuscript.

## Conflict of Interest

The authors declare that the research was conducted in the absence of any commercial or financial relationships that could be construed as a potential conflict of interest.
